# Voluntary Stopping of Eating and Drinking in Switzerland From Different Points of View: Protocol for a Mixed-Methods Study

**DOI:** 10.2196/10358

**Published:** 2018-12-21

**Authors:** Sabrina Stängle, Wilfried Schnepp, Mirjam Mezger, Daniel Büche, André Fringer

**Affiliations:** 1 Department of Nursing Science Faculty of Health University Witten/Herdecke Witten Germany; 2 Institute of Nursing School of Health Professions Zurich University of Applied Sciences Winterthur Switzerland; 3 Palliative Centre St Gallen Cantonal Hospital St Gallen St Gallen Switzerland

**Keywords:** voluntary stopping of eating and drinking, project protocol, questionnaire, cross-sectional study, focus group interviews, explorative descriptive qualitative research, practical recommendation, palliative care

## Abstract

**Background:**

“To die with dignity” has reached the significance of a core value in democratic societies. Based on this unconditional value, people require autonomy and care. "Voluntary stopping of eating and drinking" (VSED) represents an alternative to assisted suicide because no one else is involved in the action of death fastening, even though from outside, it might be considered as an extreme form of passive euthanasia. However, there are no data available about the prevalence and frequency of either explicit VSED or the implicit reduction of food and liquid in Switzerland. The responsible and independent ethics committee of the Greater Region of Eastern Switzerland (EKOS 17/083) approved this study.

**Objective:**

The objectives of the study were to research the prevalence and frequency of different types (implicit and explicit) of VSED in Switzerland; to explore the experiences, attitudes, handling and recommendations made by palliative care experts; to develop a practical recommendation about VSED, which will be validated by experts in Delphi rounds.

**Methods:**

This protocol describes a convergent mixed-method design to answer the research questions. In the first step, a cross-sectional trilingual survey (in German, French, and Italian) will be carried out to obtain a comprehensive representative picture of VSED in Switzerland. In the second step, qualitative research will be carried out by focus group interviews with palliative care experts. The interviews will be recorded, transcribed, and analyzed using generic coding, and embedded in an explorative descriptive qualitative approach. Based on the results of the first two steps, a practical recommendation will be developed. Experts will validate the practical recommendation in Delphi rounds.

**Results:**

The enrolment was completed in summer of 2018. Data analysis is currently underway and the first results are expected to be submitted for publication in the end of 2019.

**Conclusions:**

The results of this study will provide important information about the prevalence and frequency of VSED as well as the interpretation of palliative care experts about handling VSED in daily work. Furthermore, the practice recommendation will help professionals and institutions to improve the quality of care in patients and their relatives who made the decision to fasten death by VSED.

**International Registered Report Identifier (IRRID):**

DERR1-10.2196/10358

## Introduction

### Background

“To die with dignity” is a metaphor for finding a good end to one’s own life. When asked about a “good death,” studies have shown that people name the following factors: consciously experienced death at home, without pain, in good mental condition, at the end of a long, fulfilled life, and after having arranged “last things”. For many people, “death with dignity” includes autonomy, control, and self-determination [1-4].

Even under comprehensive, high-quality palliative care, suffering during the process of dying is not always avoidable [5]. Facing unbearable pain despite palliative and therapeutic possibilities results in the wish to prematurely end one’s life. Particularly at the end of life, many aspects of suffering cannot be addressed effectively by means of symptom control or modern medical treatment [6]. Various reasons result to wishing for a premature death, for example, low quality of life, senseless suffering, fear of losing one´s independence and dignity, or the wish to control the conditions of death while maintaining self-determination [7]. Mental and spiritual suffering, symptom clusters, bleeding, open wounds, altered body image, social isolation, and loss of the meaning of life are further forms of suffering, reaching far beyond pure symptom control [6,8].

The wish to prematurely end one´s life seems to be difficult to understand for persons who have not been confronted with unbearable suffering. To induce premature death, persons turn to organizations offering assisted suicide, which is possible in Switzerland for people with terminal diseases whose end of life is imminent [9]. However, taking barbiturates is not a viable option for everyone. Similar to that in the Netherlands [10,11], people are looking for other options, including the decision to engage in “voluntary stopping of eating and drinking” (VSED). VSED is the clearly stated desire of a person to abstain from eating and drinking to deliberately end his life prematurely. It takes 7 to 21 days until the process of dying is initiated. It is only interrupted when the person has second thoughts and starts eating again. VSED seems to be an ethical way to end unbearable suffering in a self-determined way [12-14]. This does not seem to be very common for several reasons [15]. Nevertheless, owing to lacking popularity of VSED or complete rejection of prematurely inflicted death, moral conflicts can arise on the part of medical and nursing professionals [16-18]. Food intake is associated with high social value and symbolizes social participation [19]. For this reason, family members may interpret VSED as refusal or deficit of professional care [12]. However, patients, particularly those in advanced stages of an oncological condition, explicitly express their wish for premature or assisted death to doctors and nurses [20]. Furthermore, there is an unverified assumption that VSED leads to intensified, additional suffering for patients [12]. Even in the scientific community, VSED is rarely taken into account as an option. These aspects can be regarded as possible reasons for rare scientific research on VSED.

Next to the phenomenon of VSED as a clearly expressed desire of a person to prematurely end her or his life, there are other forms. If a person refuses to eat and drink but is unable to explain or does not want to talk about it, this is called implicit or unspoken (V)SED [21], which is not uncommon [22]. This poses a major challenge for professionals and relatives to first recognize the refusal of eating and drinking and to explore the reasons behind it.

### Aims and Research Questions

This study aims to investigate the prevalence and frequency of VSED in Switzerland. In detail, the occurrence of implicit (V)SED and explicit VSED as well as the experiences, attitudes, handlings, and recommendations made by palliative care experts deserve study. Based on these results and in addition to previous and updated work, a practice recommendation shall be developed for professionals in specialized palliative care. The practice recommendation will be specified and evaluated by experts in Delphi rounds. For this study, the following research questions are essential.

#### Part I

- How frequent and prevalent is the implicit (V)SED and explicit VSED in palliative care throughout Switzerland?
- What kinds of experiences, attitudes, handlings, and recommendations regarding VSED exist when comparing palliative care experts?

#### Part II

- What is the perception and interpretation of the implicit (V)SED and explicit VSED in Switzerland for experts in palliative care in their daily work?
- What interpretations do the experts in palliative care drag from the experiences, attitudes handlings, and recommendations in their daily work?

#### Part III

- What information in the form of a practice recommendation based on the findings in Parts I and II and available international research literature can be gathered regarding VSED?

## Methods

### Background

The theoretical framework is based on the SENS-model of Eychmüller [23]. This approach structures palliative care problems at the end of life with the aims of palliative care: self-efficacy, self-determination, security, and support. These aims including the World Health Organization definition is the basis of the SENS-model, which is clustered into 4 main areas: symptom management, decision making, network, and support [23]. To answer the research questions, a convergent mixed- methods design [24] will be used to pursue the research questions in Parts I and II. Quantitative data will be collected through a cross-sectional trilingual survey (in German, French, and Italian). This will be used to obtain a comprehensive representative picture of the prevalence and frequency, meaning, handling, and recommendations of VSED in Switzerland [25]. Qualitative data will be collected using focus group interviews to obtain palliative care experts’ meaning and interpretation of experiences, attitudes, handlings, and recommendation in their daily work. After completion of Part I and Part II, quantitative and qualitative data are going to be integrated and discussed.

The reason for choosing this mixed-methods design is based on the integrity of the data, which influence each other. Both instruments are going to be developed at the same time. With this approach, quantitative data can be compared and integrated with qualitative data and vice versa. This brings the advantage that possible inaccuracies are reduced by different viewpoints.

Subsequently, a practice recommendation will be developed based on the survey results (Part I) and focus group interviews (Part II) as well as the current literature. Furthermore, it will be subsequently validated in expert discussion rounds based on the Delphi method [26].

### Part I: Survey

#### Instrument

A standardized evidence-based questionnaire is going to be developed, tested, and translated into French, Italian, and additionally into English for reasons of publication. The development of the questionnaire [27-31] is planned to be based on a literature update of the review of Ivanović, Büche [10]. The questionnaire will be tested by 10-20 experts in a standard pretest [29,32-34] and validated by 15-30 experts through the content validity index of Polit, Beck [35]. The forward and backward translation will be carried out for each language by 2 independent translators, subsequently aiming for a consensus through a consultant based on the Standard Linguistic Validation Process of Mapi-Institute [36].

#### Recruitment and Sampling

A representative sample of palliative care experts, employees in ambulatory care service, Long-Term Care, and general practitioners will be sought for in each case. Access to palliative care experts is first sought through professional association.

- Outpatient care: The employees are informed about the questionnaire via Associations Spitex priveé Suisse [37] and Spitex, a nonprofit professional association for outpatient care [38] via email and invited to participate.
- The employees in Long-Term Care should be contacted through CURAVIVA—Association Homes and Institutions Switzerland [39] via email for answering the questionnaire.
- The physicians should be reached through Foederatio Medicorum Helveticorum, Association of Swiss doctors [40].

Two reminders are scheduled for 3-4 weeks, which are either sent again by the respective professional association or directly by the first author. Members’ mails are obtained through the professional associations’ website. The survey can be started in February and depends on the correspondence and the possible resulting requirements (eg, the introduction of specific questions at the end of the survey) of the professional associations. The recruitment of all professional associations was planned until June 2018. The survey lasted until the end of August. We expect a return rate of 20% per group. This will be as follows: Associations Spitex priveé Suisse 35 of 175 members; Spitex 85 of 426 members; CURAVIVA 260 of 1302 members; and Foederatio Medicorum Helveticorum 282 of 1411 members.

#### Data Collection and Analysis

Questback will be used for the survey of the target groups. This is a state-of-the-art software and has a high standard for Web-based surveys [25,34]. Two reminders scheduled for 3 weeks are planned to get as many answers as possible. Data analysis is conducted using SPSS Version 24 and will be supported by a statistician. The descriptive statistics will be reported in absolute and relative values with indications of mean (SD) or mode. Ordinal variables will be calculated using the Mann-Whitney U test or Kruskal-Wallis test by ranks and Spearman rho. Nominal variables will be calculated using chi-square (Fisher’s exact test) and Mann-Whitney U test or Student *t* test and metric variables using analysis of variance.

In addition to descriptive statistical analyses, methods of inferential statistics will be used [41,42]. The following hypotheses underlie the research:

- There is a difference between the 3 target groups, Outpatient Care, Inpatient Long-Term Care, and Family Physicians, and their gender in terms of occurrence, stands, and attitude [43,44].
- There are differences between younger (<50 years) and older (50 years) participants in terms of occurrence, stands, and attitude [45].
- There is a difference between participants in terms of levels of competence in palliative care in terms of occurrence, stands, and attitude [45].
- There is a difference between participants with short (<20 years) and long (≥20 years) work experience in terms of occurrence, stands, and attitude [45].
- There is a difference between attendees with and without personal experience accompanying a person with the VSED in terms of stands and attitudes [44].

### Part II: Focus Group Interviews

Qualitative data will be collected by interviewing experts of palliative care in focus group settings. Because the VSED phenomenon can lead to controversial discussions both inter- and intraprofessionally, utilizing focus groups is particularly suitable for showing the different attitudes. We hope that the participants’ possible different views will allow us to gain deeper insight into understanding their attitude. Therefore, an explorative descriptive qualitative approach [46,47] was selected, following the coding methodology of Saldaña [48]. The focus group interviews will gather information about the meaning and interpretation of experts in the prevalence and frequency of VSED as well as the experiences, attitudes handlings, and recommendations about implicit (V)SED and explicit VSED in their daily work.

#### Recruitment and Sampling

The participants of palliative care congresses and network meetings have been sought out for this purpose. These congresses are well suited to nurses and medical professionals who are often confronted with end-of-life decisions and are expected to include participants with and without experience in caring for a person during VSED. Accordingly, 5-6 groups with up to 10 participants are covered. Each group will discuss 1 of the following topics for about 50 minutes: caring for the person concerned, support of relatives, accompaniment of professionals, how to deal with implicit (V)SED, and the appropriate use of recommendations and communication about VSED.

#### Data Collection and Analysis

A moderator will perform semistructured interviews that will be digitally recorded [49,50]. According to the pragmatic rules of transcript by the approach of Flick [51], it will be carried out using MAXQDA 12. Data will be analyzed following the constant comparative method described by Glaser [52]. Codes will be built through an inductive analyzing procedure using generic coding (first and second cycles), as described by Saldaña [48]. Each new incident, which can be assigned to an already existing code, is compared with the incidents it contains. With this approach, the properties and dimensions can be developed and the code becomes a category that can be related to other categories. In the second step, possible characteristics of the participants are drawn to their statements. If, for example, the data show that participants with palliative care education tend to have a different response as participants without further training, these are specifically compared with each other in the categories. By describing the categories considering the relationship with each other, a theory is developed.

### Data Integration of Part I and Part II

In this step, the results of Part I and Part II will be integrated and discussed. If possible, quantitative data will be qualified and qualitative data will be quantified. Owing to the very different sample sizes from the survey and focus groups, it is likely that data integration will take an explanatory approach. This means that comparable statements from the quantitative and qualitative studies are listed side by side in a table and will be related to each other; for example, factors (described by the focus groups) can be identified and describe why an item (from the survey) was answered that way [24].

### Part III: Practice Recommendation

### Instrument Development

Based on the findings from Part I and Part II and especially the findings of the data integration section of Part I and Part II, a development of a practice recommendation is being considered. The aim is to set strategies for professionals to deal with implicit (V)SED and explicit VSED with patients, relatives, and interdisciplinary team members. As basis for development, international publicized articles will be updated. The developed practice recommendation will then be refined in the project team and in cooperation with the project partners.

### Delphi and Consensus Procedures

Approximately 20-30 experts and scholars from various disciplines (nursing, medicine, geriatrics, palliative experts, ethicists, and sociologists) will then validate the final practice recommendation in a minimum of 2 and if needed, several Delphi rounds until a consensus is reached. The experts are assigned the task of checking the practice recommendation for intelligibility, comprehensibility, and completeness. The experts will be acquired via the existing network of VSED. The Delphi rounds will be carried out with the survey tool QuestBack and will be conducted in English. The consensus procedure will be carried out personally to ensure the quality of the responses.

### Ethics Approval and Consent to Participate

This investigation is neither a clinical trial nor an observational study with vulnerable groups. Thus, no personal data will be collected. Participation in the study is voluntary, and the participants will be assured irreversible anonymity. Nonparticipation does not entail any disadvantages. With an accompanying letter (via email, cover letter, and information letter on the webpage), the participants will be informed in detail and made aware of the aim and purpose of the study as well as the utilization of the generated data and their personal rights. The participants of the focus group interviews will be informed about the project in advance and asked when the interview starts. The ethical approach is based on the principles of the “Declaration of Helsinki” and “informed consent.” Anonymity and respect for human dignity is guaranteed at all times during the research process. Drawing any conclusions about the respondents will not be possible at any time [53]. The responsible institutional review board of the Greater Region of Eastern Switzerland (EKOS 17/083) approved this study.

## Results

After approval of the Ethics Committee (EKOS 17/083), the Swiss Academy of Medical Sciences provided the first partial funding. As soon as the first results are available and the study phase Part III begins, the second partial funding will be planned. The funding from Ebnet-Foundation and palliacura have each paid their share. The development of the questionnaire (Phase I) started in 2016 and was completed in the spring of 2017. Contacts to the professional associations were successfully established, and the recruitment was completed by the end of August. The focus group interviews have since been completed, and the transcription of the audio files are in progress. The results are forthcoming.

## Discussion

### Strengths

In Switzerland, there is a very high willingness to become a member of professional associations. Through the participation and support of the professional associations, we are able to guarantee a Switzerland-wide survey.

### Limitations

We recognize that that a trilingual survey, despite the translation process, carries the risk that participants’ statements may be interpreted and answered differently. In addition, the results will subsequently be published in English, which translates the statements further. The participants of the study will be given the summarized results in 3 languages via the professional associations and sent via a newsletter. To ensure that the participants in the study receive the results, the participating professional associations receive the summarized results in 3 languages and make them available for internal use.

## Abbreviations

VSED: voluntary stopping of eating and drinking
